# The Impact of Vaginal Bacteria and Antimicrobial Treatment on Pregnancy Outcomes in Healthy Breeding Bitches

**DOI:** 10.3390/antibiotics15070637

**Published:** 2026-06-25

**Authors:** Alicia Rojahn, Anna Sophia Leps, Eva-Maria Packeiser, Ute Siesenop, Jutta Verspohl, Sandra Goericke-Pesch

**Affiliations:** 1 Unit for Reproductive Medicine—Clinic for Small Animals, University of Veterinary Medicine Hannover, Foundation, 30559 Hannover, Germany; alicia.rojahn@tiho-hannover.de (A.R.); anna.leps@outlook.com (A.S.L.); eva-maria.packeiser@tiho-hannover.de (E.-M.P.); 2 Institute for Microbiology, University of Veterinary Medicine Hannover, Foundation, 30173 Hannover, Germany; ute.siesenop@tiho-hannover.de (U.S.); jutta.verspohl@tiho-hannover.de (J.V.)

**Keywords:** canine reproduction, dog, breeding, reproductive microbiome, vaginal bacteria, antimicrobial resistance, antibiotics

## Abstract

**Background/Objectives:** Prophylactic antimicrobial use prior to mating in clinically healthy breeding bitches based on vaginal culture results is common despite lacking evidence for a beneficial effect on fertility. Thus, this practice is questionable due to the risk of the development of antimicrobial resistance and dysbiosis. The study aimed to investigate whether vaginal bacteria and antimicrobial treatment influence the pregnancy outcome. **Methods:** We retrospectively analyzed vaginal swab results from healthy breeding bitches prior to mating. Samples were examined using aerobic culture, and bacterial isolates were identified by MALDI-TOF. The medical records provided data on antimicrobial treatment and pregnancy outcome. **Results:** Of the 961 available samples, 467 cases had complete information about antimicrobial use and pregnancy outcome. Overall pregnancy rates did not differ significantly between antimicrobial-treated (81.7%) and untreated bitches (79.8%) (*p* = 0.6922), nor in cases with monocultures (*p* = 0.4823), high-grade bacterial growth (*p* = 0.4291), or high-grade growth of *Escherichia coli* (*p* > 0.9999) and *Streptococcus canis* (*p* = 0.711). **Conclusions:** In this study population, antimicrobial use did not improve pregnancy rates in healthy bitches, even in cases of opportunistic bacteria. No correlation between vaginal bacteria, antimicrobial use, and pregnancy outcome was identified. Based on these findings, antimicrobial treatment of clinically healthy animals as part of breeding management cannot be recommended and should be disregarded in the context of responsible antimicrobial use.

## 1. Introduction

Antimicrobial resistance (AMR) is a growing global problem and has been described by the World Health Organization as one of the greatest threats to public health [1,2]. Each use of an antimicrobial agent carries a risk of AMR development, even in cases with a clear indication and antimicrobial susceptibility testing (AST) [3,4,5]. Studies are therefore needed to identify areas where use can be minimized without compromising patient care. Animals and humans not only share the same environment but also get treated with the same antimicrobial classes. Thus, according to the One Health approach, AMR bacteria can be transmitted bidirectionally between humans and animals [6,7,8]. Consequently, antimicrobial treatment in animals also has implications for human health [6,7,8]. Animals can act as reservoirs for AMR bacteria and resistance genes, and due to their close contact with humans, companion animals in particular may serve as a source of transmission [9,10]. 

Canine breeding represents a particular concern regarding the development of AMR. Several studies have reported high resistance rates in breeding dogs, indicating increased antimicrobial use in breeding kennels [11,12,13,14,15]. In canine reproduction, it is common practice to collect vaginal swabs from clinically healthy bitches prior to mating for bacteriological examination [16]. This often results in antimicrobial treatment based on the assumption that certain bacteria in the vaginal tract are associated with unsuccessful breeding [16]. However, the physiological vaginal microbiota predominantly consists of opportunistic bacteria [17,18,19,20,21,22,23,24,25,26,27], including *Escherichia* (*E.*) *coli* and *Streptococcus* (*Strep.*) *canis*/beta-hemolytic streptococci (BHS). These are often considered alarming by breeders and veterinarians due to their occasional association with certain disease processes such as pyometra, abortion, and neonatal mortality [18,27,28,29,30,31]. In a previous questionnaire-based study, we demonstrated that the administration of antimicrobials to clinically healthy breeding bitches remains widely practiced [32]. Moreover, detection of monocultures or high-grade bacterial growth frequently serves as the basis for antimicrobial use in canine breeding [32], as both findings have traditionally been interpreted as signs of bacterial overgrowth. Nevertheless, several studies using conventional culture examination have shown that monocultures and high-grade bacterial growth are regularly detected in healthy bitches [22,25,33]. Consequently, neither the presence nor the composition of vaginal bacteria reliably distinguishes healthy from diseased dogs [19,23,24,34,35]. It is hypothesized that antimicrobials should be administered only in the presence of clinical symptoms, such as signs of inflammation detected on gynecological examination, regardless of the bacterial findings [21,23,24,33,36]. This hypothesis is supported by recent culture-independent methods using 16s-rRNA-sequencing, which indicate that true monocultures in the vaginal tract likely do not exist [36,37,38,39,40]. Conventional culture methods overestimate easily cultivable bacteria, while they underestimate difficult-to-culture bacteria [41,42,43,44,45].

Uncertainties in interpreting vaginal bacterial findings due to limited evidence regarding the effect on canine fertility commonly result in prophylactic prescription (“to be on the safe side”), often postulated to be driven by breeder expectations [32]. However, antimicrobial treatment not only promotes AMR but also dysbiosis. This association is well known for the intestinal microbiome and its health in humans [46,47,48] and dogs [49,50,51]. As shown in women, a healthy vaginal microbiome is equally critical for genital health and fertility [52,53,54,55]. Antimicrobials can disturb the balance of the microbial environment by eliminating nutrient-competing commensals [56], thereby enabling the proliferation of facultative pathogens and resistant bacteria, with adverse effects on fertility [57].

Apart from one study using next-generation sequencing to correlate the reproductive microbiome with fertility data [37], studies on the impact of vaginal bacteria and antimicrobials on fertility are lacking. Therefore, we evaluated pregnancy rates of healthy bitches in relation to vaginal bacteriological culture findings and antimicrobial treatment, aiming to assess the effect on pregnancy outcome.

## 2. Results

**Figure 1:**
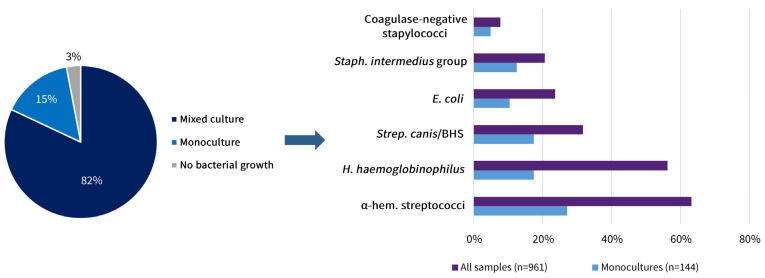
Left: Results of bacterial cultures from all vaginal swabs (n = 961), classified as monoculture, mixed culture, or no bacterial growth. Right: Most frequently isolated bacteria from all vaginal swabs (n = 961) and from monocultures (n = 144). Results are shown as a percentage of samples. BHS: beta-hemolytic streptococci.

A total of 961 samples were analyzed, yielding 54 different bacterial species. The bacterial isolates were grouped according to their taxonomic classification and cultural characteristics (Table 1). Of all samples, 82.0% (n = 788) were mixed cultures, 15.0% (n = 144) were monocultures, and 3.0% (n = 29) showed no bacterial growth. Mixed cultures were defined as samples with at least two different bacterial isolates and ranged from predominantly two (n = 424; 53.8%) up to six (n = 2; 0.3%) bacteria per sample. The most frequently isolated bacteria/bacterial groups were alpha-hemolytic (α-hem.) streptococci (n = 607; 63.2%), *Haemophilus* (*H*.) *haemoglobinophilus* (n = 540; 56.2%), *Strep. canis*/BHS (n = 305; 31.7%), *E. coli* (n = 227; 23.6%), *Staphylococcus* (*Staph*.) *intermedius* group (n = 198; 20.6%), and coagulase-negative staphylococci (n = 74; 7.7%). These bacteria/bacterial groups were also the most common monocultures (Figure 1).

**Figure 2:**
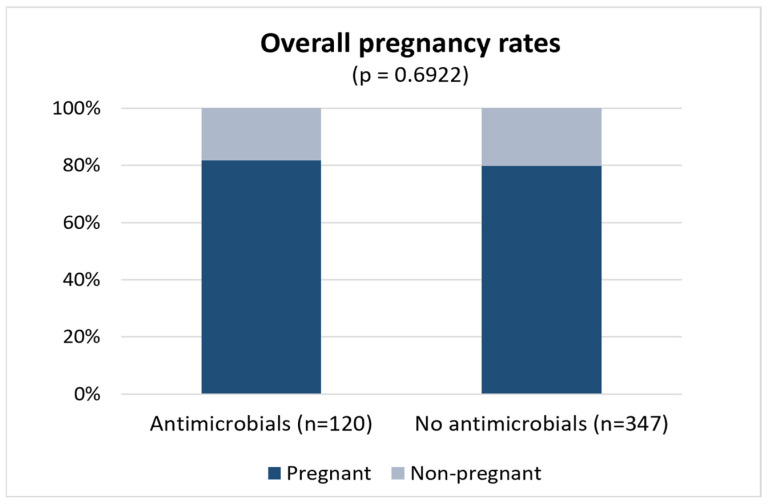
Overall pregnancy rates in all cases (n = 467) treated with antimicrobials (n = 120) or left untreated (no antimicrobials, n = 347). Results are shown as percentages of pregnant and non-pregnant bitches according to antimicrobial use.

Complete information about antimicrobial use and pregnancy outcomes was available for 467 of the 961 cases. The remaining cases were excluded from the pregnancy outcome analysis due to missing follow-up data, as some owners did not return for a pregnancy ultrasound and/or could not be contacted retrospectively to obtain outcome information. In these 467 cases, there were no significant differences in overall pregnancy rates between antimicrobial-treated (98/120, 81.7%) and untreated bitches (277/347, 79.8%) (*p* = 0.6922) (Figure 2). In 70 cases with known pregnancy outcome, the animals had confirmed bacterial monocultures. Of these, 15 bitches had monocultures of *E. coli* (high-grade, n = 4; intermediate-grade, n = 1), *Strep. canis* (high-grade, n = 5; intermediate-grade, n = 1), *Staph. intermedius* group (intermediate-grade, n = 1), *H. haemoglobinophilus* (high-grade, n = 1), *Klebsiella pneumoniae* (high-grade, n = 1), and *Mycoplasma* sp. (low-grade, n = 1) and were treated according to the results of the AST. The dog with *Mycoplasma* sp. monoculture was treated with enrofloxacin according to general recommendations [58]. Among these treated dogs, 13 became pregnant, whereas two did not conceive (*Strep. canis*, intermediate-grade, n = 1; *Mycoplasma* sp., low-grade, n = 1). Furthermore, pregnancy was confirmed in 41 of 55 untreated animals with confirmed monocultures. In these 41 bitches that did not receive antimicrobial treatment and became pregnant, monocultures of *Strep. canis* (high-grade, n = 3; low-grade, n = 1) and *E. coli* (intermediate-grade, n = 1), as well as *Staph. intermedius* group (n = 6), α-hem. streptococci (n = 18), *Bacillus* spp. (n = 3), and *H. haemoglobinophilus* (n = 9), were identified. In the remaining 14 bitches that did not receive antimicrobial treatment, but did not become pregnant, monocultures of α-hem. streptococci (intermediate-grade, n = 3; low-grade, n = 3), *Enterococcus faecalis* (low-grade, n = 1), *H. haemoglobinophilus* (low-grade, n = 1), coagulase-negative staphylococci (low-grade, n = 1), *Staph. intermedius* group (low-grade, n = 3), BHS (low-grade, n = 1), and *E. coli* (low-grade, n = 1) were confirmed. An overview summarizing the pregnancy outcomes of the detected bacterial monocultures is provided in Table 2. Pregnancy rates did not differ significantly between treated and untreated dogs (*p* = 0.4923). Additionally, Table 2 shows the pregnancy rates for mixed cultures and cultures with no bacterial growth in relation to antimicrobial treatment. Since no antimicrobial treatment was administered in cases without bacterial growth, only cases with mixed cultures were included in the statistical analysis, in which pregnancy rates did not differ significantly between treated and untreated animals (*p* > 0.9999).

**Figure 3:**
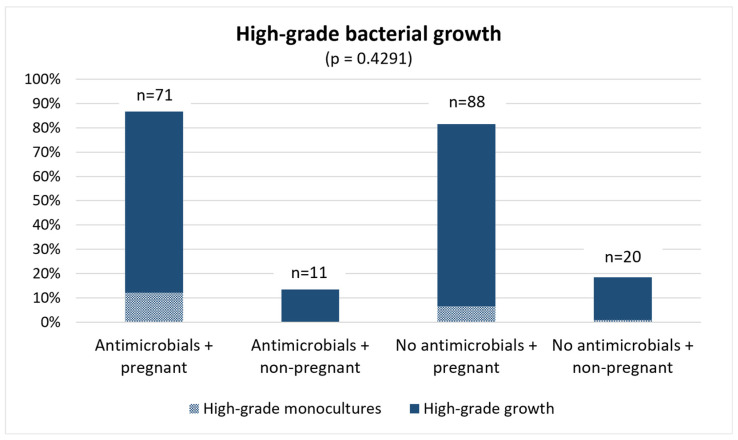
Pregnancy outcomes in cases with high-grade bacterial growth (n = 190) are shown as percentages, differentiating whether animals had been treated or not (antimicrobials n = 82, no antimicrobials n = 108) and whether they became pregnant or not. Additionally, the proportion of monocultures and the number of cases (n) for each group are given.

Figure 3 shows the pregnancy rates for cases with high-grade bacterial growth (n = 190), a finding that often serves as a basis for antimicrobial treatment in healthy breeding bitches [32], with 18 cases representing high-grade monocultures. No significant differences were observed in pregnancy rates between bitches with high-grade bacterial growth that received antimicrobials (pregnant/all treated: 71/82 bitches) and those that did not (pregnant/all untreated: 88/108 bitches) (*p* = 0.4291) (Figure 3). Among the untreated high-grade monocultures (untreated/all high-grade monocultures: 8/18 animals), seven resulted in pregnancy, with high-grade monocultures identified as *Strep. canis* (n = 3), *H. haemoglobinophilus* (n = 2), α-hem. streptococci (n = 1), and *Staph. pseudintermedius* (n = 1). Only one untreated case with a high-grade monoculture of *Enterococcus faecalis* did not result in pregnancy.

Table 3 provides details on pregnancy outcomes for detected high-grade growth of *E. coli* and *Strep. canis*. Concerning high-grade growth of *E. coli* (n = 36), pregnancy was confirmed in 8/9 untreated and 23/27 treated bitches, with no significant differences between groups (*p* > 0.9999). Similarly, for high-grade growth of *Strep. canis* (n = 63), pregnancy was observed in 17/19 untreated dogs, including three cases with high-grade monocultures. In comparison, 37/44 treated dogs became pregnant (*p* = 0.711).

The results of the statistical analyses of the primary comparisons, including overall cases, monocultures, high-grade bacterial growth, and high-grade growth of *E. coli* and *Strep. canis*, indicated no statistically significant associations (Table 4). Furthermore, Table 5 shows the association between pregnancy outcome and potential confounders, including antimicrobial use, age (≤5/≥5 years), type of culture (mixed culture/monoculture), presence of high-grade growth of *E. coli* or *Strep. canis*, type of mating (natural mating, fresh or frozen semen insemination), and parity (primiparous/multiparous). None of the evaluated variables showed a statistically significant association. Older animals showed a tendency towards lower odds of pregnancy, as did animals that received antimicrobials. The detection of high-grade growth of *E. coli* and *Strep. canis* was associated with slightly higher odds of pregnancy. Regarding the type of mating, natural mating and frozen semen insemination tended to show lower odds of pregnancy compared to artificial insemination with fresh semen. The type of culture and parity revealed no relevant association.

## 3. Discussion

Several studies have compared the vaginal microbiota of healthy bitches and those with fertility problems without detecting compositional differences [18,19,24,34]. However, this is the first study to directly investigate vaginal bacterial culture findings, antimicrobial use, and pregnancy outcome in dogs.

Unlike Gropetti et al., who only compared positive cultures and cultures without bacterial growth in relation to pregnancy outcomes and reproductive diseases, and suggested that cultures with bacterial growth might even be associated with a favorable prognosis [21], our study evaluated specific bacterial findings as well as the grade of growth while accounting for potential confounders. Similarly, Leps et al. assessed the association between bacteria and pregnancy outcome; however, they used 16S-rRNA-sequencing with a particular focus on *Mycoplasma* spp. The authors indicated that the influence of the microbiome on fertility may be overestimated [37]. Against this background, our study closes an important gap by evaluating the impact of routinely applied culture-based vaginal bacterial results and antimicrobial treatment on pregnancy outcomes in healthy breeding bitches, focusing on specific bacterial findings that frequently result in non-evidence-based antimicrobial treatment.

The results confirm that the physiological vaginal microbiota commonly include opportunistic bacteria such as *Strep. canis*/BHS, *E. coli*, and bacteria of the *Staph. intermedius* group. Furthermore, 15% of samples yielded monocultures, which is in line with earlier studies [22,25,26,33], emphasizing that monocultures can be regularly detected in clinically healthy animals. The fact that 3% of samples showed no bacterial growth highlights that examination methodology strongly influences the outcome, since the vagina is not a sterile environment [36].

A total of 467 cases with complete information on antimicrobial use and pregnancy outcome were available for analysis. Statistical comparison of bitches treated with antimicrobials and those left untreated revealed no significant differences: In both groups, approximately 80% of dogs became pregnant. Direct comparison between treated and untreated animals is, however, limited by various potential confounders that could influence the pregnancy outcome. In this study population, antimicrobial use showed a tendency for lower odds of pregnancy, while accounting for age, type of culture, high-grade growth of *E. coli* and *Strep. canis*, type of mating, and parity. Nevertheless, results were not statistically significant, and a possible history of infertility of the dogs—guiding the decision for antimicrobial treatment based on vaginal bacterial culture findings—was not considered. In clinical practice, mixed cultures are generally interpreted as physiological and of no clinical concern. In contrast, findings that still frequently result in antimicrobial therapy in clinically healthy breeding bitches are monocultures and high-grade bacterial growth [32]. We therefore directly compared cases with these findings. Again, no significant differences or associations were observed, neither in cases with monocultures nor in those with high-grade bacterial growth. Even bitches with high-grade monocultures conceived without antimicrobial treatment. Furthermore, no specific bacterial species associated with infertility could be identified among the non-pregnant dogs. As anticipated in previous studies [23,24,25,26], our results confirm that monocultures and high-grade growth do not necessarily reflect true bacterial overgrowth in clinically healthy bitches. These findings are more likely attributable to limitations in culture-based diagnostics related to sampling and culturing techniques. For example, the use of a speculum, the sampling site [59,60], and storage conditions [61,62] can influence bacterial growth and, therefore, affect microbiological outcomes. Moreover, new culture-independent methods have clearly demonstrated that true monocultures do not exist in the canine vagina [36,37,38,39,40], since they provide a more comprehensive picture of the microbiome [41,42,43,44,45].

In addition to that, the role of *E. coli* and *Strep. canis*/BHS is often controversially discussed by veterinarians and breeders. Their involvement in specific reproductive disorders is well recognized [18,27,28,29,30,31]. Consequently, their detection in clinically healthy bitches frequently leads to prophylactic antimicrobial treatment. We therefore specifically analyzed cases with high-grade growth of *E. coli* and *Strep. canis*. No significant differences in pregnancy rates were detected between treated and untreated bitches. Moreover, the detection of high-grade growth of *E. coli* and *Strep. canis* was even associated with slightly higher odds of pregnancy in this population, albeit not statistically significant. These findings suggest that prophylactic antimicrobial treatment cannot be justified from a perspective of responsible antimicrobial use in healthy breeding bitches. It should be noted that the data were collected between 2016 and 2025, and that diagnostic and therapeutic recommendations have evolved over time. Until a few years ago, it was common practice to treat bitches with such culture results before mating, which explains the comparatively high number of treated cases in our dataset. More recent studies, however, suggest that even in cases of high-grade monocultures, antimicrobials should not be administered in the absence of clinical signs [25,26]. Our results support this hypothesis, particularly as one key finding was that dogs with high-grade monocultures of *Strep. canis* conceived successfully without antimicrobial treatment. These observations align with those of Tesin et al., who identified BHS and *E. coli* among the most frequently isolated bacteria in pregnant bitches [63].

In recent years, awareness of prudent antimicrobial use has increased, especially among veterinarians [64,65,66]. Nonetheless, canine breeding remains an area of particular concern regarding AMR. A recent study documented a higher prevalence of extended-spectrum beta-lactamase-producing *E. coli* and methicillin-resistant *Staphylococcus pseudintermedius* in breeding dogs in Italy compared to pets without breeding use [14]. Beyond AMR development, antimicrobials can have additional detrimental effects on the animal. Systemic antimicrobial therapy can affect all microbial ecosystems of the body, such as the intestine, skin, ear canal, oral and nasal cavities, potentially inducing dysbiosis and predisposing to medical conditions [67]. This should be considered when breeders request antimicrobial treatment and/or veterinarians prescribe or feel pressured to prescribe antimicrobials to avoid being blamed for unsuccessful breeding [68,69,70]. Some owners and veterinarians are unaware of the potential negative consequences and believe that antimicrobial treatment is harmless [68,70,71,72]. However, antimicrobial use in breeding bitches can disrupt the vaginal microbiota and facilitate overgrowth of potentially pathogenic and resistant organisms by eliminating competing commensals [56,67]. This was demonstrated already more than 30 years ago by Ström et al., where *Mycoplasma* spp. and *E. coli* became apparent after antimicrobial treatment [57]. Future studies should investigate the impact of antimicrobial treatment on the vaginal microbiota using follow-up samples. In human medicine, a disturbed vaginal flora has been associated with impaired fertility and adverse genital health outcomes [52,53,54,55]. Although our study did not identify a negative effect of antimicrobial use on pregnancy outcome, the opportunistic nature of the vaginal bacteria underlines that breeding management should focus on strengthening the immune system and optimizing housing and hygiene conditions, rather than relying on prophylactic antimicrobial administration. This concept is supported by data in a study by Rocha et al., showing that *E. coli* strains isolated from the vagina are similar to those causing pyometra, but that these strains generally exhibit low intrinsic virulence, making host factors such as immune status crucial for disease development [73]. As vaginal isolates already display various resistances [21,63,73,74], further exacerbation of the resistance situation could pose a serious threat to diseased dogs in terms of treatment efficacy. Another negative effect of antimicrobial treatment that is directly relevant to breeding is its impact on the sexual attractiveness of the bitch to the stud dog. It has been demonstrated that untreated bitches are significantly more attractive to males than those treated with antimicrobials, presumably due to the elimination of vaginal bacteria and the resulting alteration of pheromonal signaling [75].

Considering the increasing AMR crisis, alternative strategies to antimicrobials are receiving growing attention. Probiotic approaches aimed at strengthening the vaginal microbiota through competitive and antimicrobial-active bacteria have been explored [76,77,78]. However, lactic acid bacteria commonly used as probiotics are not typical members of the canine vaginal microbiota, and oral administration appears to have no direct effect on the vaginal flora [79,80], although indirect beneficial effects on its composition have been suggested [80]. These may be mediated by improved gut microbiome stability and antioxidative properties, which are known to be important for overall immunity and health [81] and were already described as beneficial for semen quality in stud dogs [82].

This study has some limitations; breeders may have administered antimicrobials without informing the attending veterinarian. However, to the best of our knowledge, we were the only treating veterinarians, and the owners followed our recommendations. Due to the large variety of breeds included and the incomplete availability of litter size data, evaluation of litter size was beyond the scope of this study. Moreover, the retrospective study design did not allow for fully standardized conditions. As a result, other factors that may have influenced treatment decisions and pregnancy outcomes could only be controlled to a limited extent. At the same time, this underlines that before initiating antimicrobial treatment for suspected sub- or infertility, all other potential causes of unsuccessful breeding, such as timing of mating, type of mating (natural mating, insemination), and semen quality, should be thoroughly investigated. It should be considered that repeated records from the same bitch across different years may have introduced bias, although all animals were clinically healthy. It should also be noted that despite sampling from the cranial vagina using a sterile tube speculum, contamination with fecal bacteria cannot be completely ruled out due to the close anatomical proximity to the perianal region. Future studies should be conducted prospectively under more standardized conditions and include additional parameters such as resorptions and litter sizes. Nevertheless, to the best of our knowledge, this is the first study to systematically investigate the relationship between vaginal bacterial culture findings, antimicrobial use, and pregnancy outcomes in bitches. Strengths of the present study include the large number of cases and the standardized sampling procedure within a single clinic. In addition, antimicrobial treatments were consistently prescribed based on AST, which largely rules out treatment failure due to the use of ineffective drugs as a cause for non-pregnancy.

## 4. Materials and Methods

### 4.1. Study Design and Sample Collection

We retrospectively analyzed bacteriological culture results of vaginal swabs collected from clinically healthy breeding bitches between January 2016 and September 2025. The dogs were presented at the Unit for Reproductive Medicine, University of Veterinary Medicine Hannover, Hannover, Germany, for a breeder-requested routine bacteriological vaginal swab examination prior to mating. All dogs underwent a general and gynecological examination, including a vaginoscopy and vaginal cytology for the detection of pathological conditions and cycle staging [59,83]. Only anatomically normal bitches in proestrus and estrus with breeding purposes and without any signs of disease were included in the study. Since all swabs were collected within the same clinic, a standardized sampling procedure was ensured. After the external gynecological examination and cleaning of the vulva with a dry towel, a sterile tube speculum (Model “Hannover”, Wirtschaftsgenossenschaft Deutscher Tierärzte eG, Garbsen, Germany) was inserted into the vagina to enable sterile sampling from the cranial vagina [59]. A sterile cotton swab (Meditip Clean Cotton tips 230 mm, servoprax GmbH, Wesel, Germany) was rotated on the dorsal mucosa through the vaginoscope and transferred into sterile Amies medium (Amies clr, Meus s.r.l., Piove di Sacco, Italy) for aerobic culturing. Subsequent information about antimicrobial treatment and pregnancy outcome was obtained from the medical records. Since we were the responsible veterinarians, to the best of our knowledge, the owners followed our treatment recommendations. If antimicrobials were prescribed, this was based on antimicrobial susceptibility testing. The antimicrobial classes used were beta-lactams, trimethoprim–sulfonamides, cephalosporins, and fluoroquinolones. Treatment duration ranged from 5 to 10 days and was completed before mating. However, the exact timing relative to mating varied. The animals included in the study were of various breeds (n = 86) and ages (mean 4.25 ± 1.81 years). Additional data were available for a subset of dogs: Bitches were either primiparous (n = 147) or multiparous (n = 175), and the type of mating was recorded as natural mating (n = 205) or artificial insemination (AI) using fresh (n = 70) or frozen semen (n = 21). Data analysis was descriptive, and Microsoft Excel^®^ (Version 2601, Microsoft Corporation, Redmond, WA, USA) was applied for data analysis and the graphical presentation. Cases with monoculture, high-grade bacterial growth, and high-grade growth of *E. coli* and *Strep. canis* were chosen for more detailed analysis due to their special concern in canine reproduction.

### 4.2. Bacteriological Culture Examination

All samples were transported to the Institute for Microbiology, University of Veterinary Medicine Hannover, for aerobic bacteriological examination. One sample was processed by the same laboratory member. The swabs were streaked onto Columbia Agar with 5% sheep blood (Oxoid GmbH, Wesel, Germany), Gassner Medium (Oxoid GmbH, Wesel, Germany), and Staphylococcus Streptococcus Selective Agar (Institute for Microbiology, University of Veterinary Medicine Hannover, Hannover, Germany). The plates were incubated aerobically at 36 °C for 48 h. Additionally, each swab was incubated in nutrient broth (Institute for Microbiology, University of Veterinary Medicine Hannover, Hannover, Germany) at 36 °C overnight, then streaked again onto Columbia Agar with 5% sheep blood, Gassner Medium, and Staphylococcus Streptococcus Selective Agar, and incubated at 36 °C for 48 h. After 24 h of incubation, cultures were first examined for bacterial growth, and after 48 h, all cultures were re-evaluated. Grown bacteria were mainly identified at the species level using matrix-assisted laser desorption/ionization-time of flight mass spectrometry (MALDI-TOF MS) (Microflex LT/SH, Bruker Daltonics GmbH & Co. KG, Bremen, Germany) with Biotyper^®^ Library (Version 13, Bruker Daltonics GmbH & Co. KG, Bremen, Germany) [84]. In some cases, *Mycoplasma* spp. were able to grow on the media described above. Bacterial growth was semi-quantitatively classified as low-grade, intermediate, or high-grade according to the extent of macroscopic culture growth. AST was performed using the broth microdilution method.

### 4.3. Statistical Analysis

Data presentation is descriptive. Cases with available information on antimicrobial treatment and pregnancy outcome were included for further statistical analysis using GraphPad Prism^®^ (Version 10.0.2, GraphPad Software, Inc., Boston, MA, USA). To determine whether a significant difference in pregnancy rates between antimicrobial-treated and untreated dogs existed, Fisher’s Exact test was performed. Furthermore, pregnancy outcomes of cases with bacterial monocultures, high-grade bacterial growth, and high-grade growth of *E. coli* and *Strep. canis* in relation to antimicrobial use were tested for significance. A *p*-value of *p* < 0.05 was considered statistically significant. The effect size was estimated by the odds ratio (OR) with corresponding 95% confidence intervals (CI), using the Baptista–Pike method. Furthermore, to assess factors associated with pregnancy outcome, a multivariable logistic regression analysis was performed. Variables included antimicrobial use, type of culture (mixed culture/monoculture), age (≤5/>5 years), presence of high-grade growth of *E. coli*, presence of high-grade growth of *Strep. canis*, type of mating (natural/artificial insemination (AI) with fresh semen/AI with frozen semen), and parity (primiparous/multiparous). The reference category consisted of bitches without antimicrobial treatment, aged ≤5 years, mixed culture result, no high-grade growth of *E. coli* and *Strep. canis*, AI with fresh semen, and primiparous animals. Regression coefficients (ß) and adjusted OR with 95% CI were calculated.

## 5. Conclusions

This study provides the first evidence that vaginal bacterial culture findings, including monocultures and high-grade bacterial growth, are not associated with impaired pregnancy outcomes in clinically healthy breeding bitches. In this retrospective study population, antimicrobial use in healthy dogs was not associated with improved pregnancy rates, even in cases where opportunistic pathogens were detected. This further supports that antimicrobial treatment of clinically healthy animals as part of the breeding management is not recommended and is not aligned with the principles of responsible antimicrobial use.

## Figures and Tables

**Table 1:** Grouping of isolated bacteria according to their taxonomic classification and cultural characteristics.

Bacterial Group	Isolated Bacteria
Coagulase-negative staphylococci and other Staphylococcaceae	Coagulase-negative staphylococci *, *Macrococcus canis*, *Macrococcus caseolyticus*
*Staphylococcus intermedius* group	*Staph. intermedius*, *Staph. pseudintermedius*, *Staph*. *intermedius* group *
Beta-hemolytic streptococci	*Strep. canis*, *Strep. dysgalactiae*, *Strep. dysgalactiae* ssp. *equisimilis*, BHS *
*Escherichia coli*	*E. coli (hemolytic), E. coli (anhemolytic)*
*Enterococcus* species	*Enterococcus canintestini*, *Enterococcus canis*, *Enterococcus casseliflavus*, *Enterococcus faecalis*, *Enterococcus faecium*, *Enterococcus raffinosus*, *Enterococcus hirae*, *Enterococcus* spp. *
Enterobacterales (excl. *E. coli*)	*Citrobacter koseri*, coliform bacteria *, *Enterobacter cloacae*, *Klebsiella aerogenes*, *Klebsiella pneumoniae*, *Pantoea agglomerans*, *Proteus mirabilis*, *Proteus* spp. *
Streptococci (excl. BHS)	α-hem. streptococci *, anhemolytic streptococci *, *Strep. minor, Strep. gallolyticus*
Pasteurellales	*Pasteurella canis*, *Pasteurella* spp. *, *Frederiksenia canicola*
*Haemophilus* spp.	*H. haemoglobinophilus*, *Haemophilus* spp. *
*Mycoplasma* spp.	*Mycoplasma canis*, *Mycoplasma* spp. *
Gram-negative non-fermenters	*Acinetobacter pittii*, *Acinetobacter radioresistens*, *Acinetobacter* spp., *Moraxella canis*, *Moraxella* spp. *, *Pseudomonas aeruginosa*, non-fermenters *, *Streptobacillus* spp. *
Gram-positive rods	*Bacillus* spp. *, *Actinomyces canis*, *Actinomyces hyovaginalis*, *Actinomyces urogenitalis*, *Actinomyces* spp. *, *Arcanobacterium canis*, *Lactobacillus* spp. *, coryneform bacteria *

* No further characterization performed.

**Table 2:** Pregnancy outcomes of cases with bacterial monocultures, mixed cultures, and no bacterial growth in relation to antimicrobial use.

	Pregnant	Non-Pregnant
Monoculture (n = 70) (*p* = 0.4923)	Antimicrobials: 13/15 No antimicrobials: 41 */55	Antimicrobials: 2/15 No antimicrobials: 14/55
Mixed culture (n = 390) (*p* > 0.9999)	Antimicrobials: 85/105 No antimicrobials: 229/285	Antimicrobials: 20/105 No Antimicrobials: 56/285
No bacterial growth (n = 19)	No antimicrobials: 18/19	No antimicrobials: 1/19

* Included *Strep. canis* (high-grade, n = 3; low-grade, n = 1) and *E. coli* (intermediate-grade, n = 1).

**Table 3:** Pregnancy outcomes of cases with high-grade growth of *E. coli* and *Strep. canis* in relation to antimicrobial use. The *p*-values indicate that antimicrobial treatment had no significant impact on the pregnancy outcomes.

	Pregnant	Non-Pregnant
High-grade growth of *E. coli* (n = 36) (*p* > 0.9999)	Antimicrobials: 23/27 No antimicrobials: 8/9	Antimicrobials: 4/27 No antimicrobials: 1/9
High-grade growth of *Strep. canis* (n = 63) (*p* = 0.711)	Antimicrobials: 37/44 No antimicrobials: 17 */19	Antimicrobials: 7/44 No antimicrobials: 2/19

* Included high-grade monocultures (n = 3).

**Table 4:** Statistical analysis of the primary comparisons (overall cases, monocultures, high-grade bacterial growth, high-grade growth of *E. coli*, and *Strep. canis*) regarding pregnancy outcome in relation to antimicrobial use. Results are presented as *p*-value, odds ratio (OR), and 95% confidence interval (CI).

	*p*-Value	OR	95% CI
Overall cases	0.6922	1.126	0.6750–1.916
Monocultures	0.4923	2.220	0.5078–10.780
High-grade bacterial growth	0.4291	1.467	9.6664–3.161
High-grade growth of *E. coli*	>0.9999	0.958	0.0662–7.281
High-grade growth of *Strep. canis*	0.7110	1.467	0.1213–2.786

**Table 5:** Multivariable logistic regression analysis of different variables (antimicrobial use, age, type of culture, presence of high-grade growth of *E. coli* or *Strep. canis*, type of mating, and parity) regarding pregnancy outcome. Results are presented as regression coefficient (ß-coefficient) and odds ratio (OR), as well as 95% confidence intervals (CI). The reference category consisted of bitches without antimicrobial treatment, aged ≤5 years, mixed culture result, no high-grade growth of *E. coli,* and *Strep. canis*, artificial insemination (AI) with fresh semen, and primiparous animals.

	ß-Coefficient	95% CI of ß	OR	95% CI of OR
Antimicrobials	−0.2971	−1.143–0.580	0.743	0.3189–1.786
Age (>5 years)	−0.2363	−0.954–0.489	0.790	0.3850–1.631
Monoculture	0.01747	−0.911–1.079	1.018	0.4021–2.941
High-grade growth of *E. coli*	0.2472	−0.964–1.648	1.280	0.3815–5.195
High-grade growth of *Strep. canis*	0.388	−0.641–1.583	1.474	0.5269–4.868
Natural mating	−0.1171	−0.955–0.6530	0.890	0.3848–1.921
AI with frozen semen	−0.3893	−1.630–0.9343	0.678	0.1958–2.546
Multiparous	−0.06071	−0.762–0.6402	0.941	0.4666–1.897

## Data Availability

The raw data supporting the conclusions of this article will be made available by the authors on request.

## References

[1] World Health Organization Antimicrobial Resistance. https://www.who.int/news-room/fact-sheets/detail/antimicrobial-resistance.

[2] World Health Organization Ten Threats to Global Health in 2019. https://www.who.int/news-room/spotlight/ten-threats-to-global-health-in-2019.

[3] Christaki E., Marcou M., Tofarides A. (2020). Antimicrobial Resistance in Bacteria: Mechanisms, Evolution, and Persistence. J. Mol. Evol..

[4] Lee J.H. (2019). Perspectives towards antibiotic resistance: From molecules to population. J. Microbiol..

[5] Weese J.S., Giguere S., Guardabassi L., Morley P.S., Papich M., Ricciuto D.R., Sykes J.E. (2015). ACVIM consensus statement on therapeutic antimicrobial use in animals and antimicrobial resistance. J. Vet. Intern. Med..

[6] Guardabassi L., Butaye P., Dockrell D.H., Ross Fitzgerald J., Kuijper E.J. (2020). One Health: A multifaceted concept combining diverse approaches to prevent and control antimicrobial resistance. Clin. Microbiol. Infect..

[7] McEwen S.A., Collignon P.J. (2018). Antimicrobial Resistance: A One Health Perspective. Microbiol. Spectr..

[8] Monteiro H.I.G., Silva V., de Sousa T., Calouro R., Saraiva S., Igrejas G., Poeta P. (2025). Antimicrobial Resistance in European Companion Animals Practice: A One Health Approach. Animals.

[9] Pomba C., Rantala M., Greko C., Baptiste K.E., Catry B., van Duijkeren E., Mateus A., Moreno M.A., Pyörälä S., Ruzauskas M. (2017). Public health risk of antimicrobial resistance transfer from companion animals. J. Antimicrob. Chemother..

[10] Guardabassi L., Schwarz S., Lloyd D.H. (2004). Pet animals as reservoirs of antimicrobial-resistant bacteria. J. Antimicrob. Chemother..

[11] Rota A., Milani C., Drigo I., Drigo M., Corro M. (2011). Isolation of methicillin-resistant *Staphylococcus pseudintermedius* from breeding dogs. Theriogenology.

[12] Milani C., Corro M., Drigo M., Rota A. (2012). Antimicrobial resistance in bacteria from breeding dogs housed in kennels with differing neonatal mortality and use of antibiotics. Theriogenology.

[13] Rota A., Milani C., Corro M., Drigo I., Borjesson S. (2013). Misuse of antimicrobials and selection of methicillin-resistant *Staphylococcus pseudintermedius* strains in breeding kennels: Genetic characterization of bacteria after a two-year interval. Reprod. Domest. Anim..

[14] Bertero A., Corro M., Del Carro A., Spagnolo E., Milani C., Diana A., Rota A. (2025). Antimicrobial pressure in healthy breeding dogs vs household animals assessed through the resistance profile of *Escherichia coli* and coagulase positive Staphylococci. Vet. J..

[15] Milani C., Diana A., Corro M., Spagnolo E., Del Carro A., Rota A., Bertero A. (2026). Antimicrobial use in breeding kennels and antimicrobial resistance profile of *Escherichia coli* and *Staphylococcus pseudintermedius* isolated from healthy breeding bitches in Northern Italy. Front. Vet. Sci..

[16] Jeschke T. (2008). Erhebung zur Situation der Caninen Reproduktionsmedizin bei Tierärzten und Züchtern—Ein Beitrag zur Erhebung des Status quo und zur Verbesserung der Lehre auf Diesem Gebiet. Ph.D. Thesis.

[17] Olson P.N., Mather E.C. (1978). Canine vaginal and uterine bacterial flora. J. Am. Vet. Med. Assoc..

[18] Bjurstrom L. (1993). Aerobic bacteria occurring in the vagina of bitches with reproductive disorders. Acta Vet. Scand..

[19] Allen W.E., Dagnall G.J.R. (1982). Some observations on the aerobic bacterial flora of the genital tract of the dog and bitch. J. Small Anim. Pract..

[20] Watts J.R., Wright P.J., Whithear K.C. (1996). Uterine, cervical and vaginal microflora of the normal bitch throughout the reproductive cycle. J. Small Anim. Pract..

[21] Groppetti D., Pecile A., Barbero C., Martino P.A. (2012). Vaginal bacterial flora and cytology in proestrous bitches: Role on fertility. Theriogenology.

[22] Maksimovic A., Maksimovic Z., Filipovic S., Besirovic H., Rifatbegovic M. (2012). Vaginal and uterine bacteria of healthy bitches during different stages of their reproductive cycle. Vet. Rec..

[23] Golinska E., Sowinska N., Tomusiak-Plebanek A., Szydlo M., Witka N., Lenarczyk J., Strus M. (2021). The vaginal microflora changes in various stages of the estrous cycle of healthy female dogs and the ones with genital tract infections. BMC Vet. Res..

[24] Jagodka D., Kaczorek-Lukowska E., Graczyk R., Socha P. (2023). Vaginal aerobic bacteria of healthy bitches and those with fertility problems. Pol. J. Vet. Sci..

[25] Schäfer-Somi S., Lechner D., Tichy A., Spergser J. (2024). The Cultivable Bacteria Colonizing Canine Vagina During Proestrus and Estrus: A Large-Scale Retrospective Study of Influencing Factors. Animals.

[26] Leps A.S., Klein B., Schneider M., Meyer C., Soba A., Simon C., Dyachenko V., Siesenop U., Verspohl J., Goericke-Pesch S. (2024). The Canine Vaginal Flora: A Large-Cohort Retrospective Study. Vet. Sci..

[27] Graham E.M., Taylor D.J. (2012). Bacterial reproductive pathogens of cats and dogs. Vet. Clin. N. Am. Small Anim. Pract..

[28] Shambulingappa B.E., Manegar G.A., Ananda K.J. (2010). Study on aerobic bacterial flora in canine abortions. Vet. World.

[29] Fontbonne A. (2023). Causes of pregnancy arrest in the canine species. Reprod. Domest. Anim..

[30] Guerrero A.E., Stornelli M.C., Jurado S.B., Giacoboni G., Sguazza G.H., de la Sota R.L., Stornelli M.A. (2018). Vaginal isolation of beta-haemolytic *Streptococcus* from bitches with and without neonatal deaths in the litters. Reprod. Domest. Anim..

[31] Pretzer S.D. (2008). Bacterial and protozoal causes of pregnancy loss in the bitch and queen. Theriogenology.

[32] Rojahn A., Leps A.S., Goericke-Pesch S. (2025). German veterinarians asked: A cross-sectional study on microbiological examination and antimicrobial use in canine reproductive medicine. Front. Vet. Sci..

[33] Bjurstrom L., Linde-Forsberg C. (1992). Long-term study of aerobic bacteria of the genital tract in breeding bitches. Am. J. Vet. Res..

[34] Osbaldiston G.W., Nuru S., Mosiert J.E. (1972). Vaginal cytology and microflora of intertile bitches. J. Am. Anim. Hosp. Assoc. (JAAHA).

[35] Hirsh D.C., Wiger N. (1977). The bacterial flora of the normal canine vagina compared with that of vaginal exudates. J. Small Anim. Pract..

[36] Lyman C.C., Holyoak G.R., Meinkoth K., Wieneke X., Chillemi K.A., DeSilva U. (2019). Canine endometrial and vaginal microbiomes reveal distinct and complex ecosystems. PLoS ONE.

[37] Leps A.S., Packeiser E.-M., Schwens C., Stoelcker B., Doric S., Wirkner M., Walter B., Wehrend A., Kichmann V., Jung K. (2025). The canine vaginal microbiome during heat and fertility in healthy breeding dogs. PLoS ONE.

[38] Rota A., Corro M., Patuzzi I., Milani C., Masia S., Mastrorilli E., Petrin S., Longo A., Del Carro A., Losasso C. (2020). Effect of sterilization on the canine vaginal microbiota: A pilot study. BMC Vet. Res..

[39] Hu J., Cui L., Wang X., Gao X., Qiu S., Qi H., Jiang S., Li F., Yin Y. (2022). Dynamics of vaginal microbiome in female beagles at different ages. Res. Vet. Sci..

[40] Gronsfeld V., Brutinel F., Egyptien S., Porsmoguer C., Hamaide A., Taminiau B., Daube G., Van de Weerdt M.-L., Deleuze S., Noel S. (2024). Evaluation of the vaginal and urinary microbiota of healthy cycling bitches. BMC Vet. Res..

[41] Theron J., Cloete T.E. (2000). Molecular techniques for determining microbial diversity and community structure in natural environments. Crit. Rev. Microbiol..

[42] Chaudhari H.G., Prajapati S., Wardah Z.H., Raol G., Prajapati V., Patel R., Shati A.A., Alfaifi M.Y., Elbehairi S.E.I., Sayyed R.Z. (2023). Decoding the microbial universe with metagenomics: A brief insight. Front. Genet..

[43] Domrazek K., Jurka P. (2024). Application of Next-Generation Sequencing (NGS) Techniques for Selected Companion Animals. Animals.

[44] Chen P., Sun W., He Y. (2020). Comparison of the next-generation sequencing (NGS) technology with culture methods in the diagnosis of bacterial and fungal infections. J. Thorac. Dis..

[45] Banchi P., Bertero A., Gionechetti F., Corro M., Spagnolo E., Donato G.G., Pallavicini A., Rota A. (2024). The vaginal microbiota of healthy female cats. Theriogenology.

[46] Jernberg C., Lofmark S., Edlund C., Jansson J.K. (2007). Long-term ecological impacts of antibiotic administration on the human intestinal microbiota. ISME J..

[47] Ramakrishna B.S., Patankar R. (2023). Antibiotic-associated Gut Dysbiosis. J. Assoc. Physicians India.

[48] Cusumano G., Flores G.A., Venanzoni R., Angelini P. (2025). The Impact of Antibiotic Therapy on Intestinal Microbiota: Dysbiosis, Antibiotic Resistance, and Restoration Strategies. Antibiotics.

[49] Pilla R., Suchodolski J.S. (2019). The Role of the Canine Gut Microbiome and Metabolome in Health and Gastrointestinal Disease. Front. Vet. Sci..

[50] Pilla R., Gaschen F.P., Barr J.W., Olson E., Honneffer J., Guard B.C., Blake A.B., Villanueva D., Khattab M.R., AlShawaqfeh M.K. (2020). Effects of metronidazole on the fecal microbiome and metabolome in healthy dogs. J. Vet. Intern. Med..

[51] Kim S.J., Chung H.C., Park S.Y., Lee J.M., Han J.H. (2025). Beneficial effects of probiotics on dysbiosis of gut microbiota induced by antibiotic treatment in healthy dogs. Res. Vet. Sci..

[52] Garcia-Velasco J.A., Menabrito M., Catalan I.B. (2017). What fertility specialists should know about the vaginal microbiome: A review. Reprod. Biomed. Online.

[53] Saraf V.S., Sheikh S.A., Ahmad A., Gillevet P.M., Bokhari H., Javed S. (2021). Vaginal microbiome: Normalcy vs dysbiosis. Arch. Microbiol..

[54] Chen X., Lu Y., Chen T., Li R. (2021). The Female Vaginal Microbiome in Health and Bacterial Vaginosis. Front. Cell. Infect. Microbiol..

[55] Ughade P.A., Shrivastava D., Chaudhari K. (2024). Navigating the Microbial Landscape: Understanding Dysbiosis in Human Genital Tracts and Its Impact on Fertility. Cureus.

[56] Leshem A., Liwinski T., Elinav E. (2020). Immune-Microbiota Interplay and Colonization Resistance in Infection. Mol. Cell.

[57] Ström B., Linde-Forsberg C. (1993). Effects of ampicillin and trimethoprim-sulfamethoxazole on the vaginal bacterial flora of bitches. Am. J. Vet. Res..

[58] Gautier-Bouchardon A.V. (2018). Antimicrobial Resistance in *Mycoplasma* spp.. Microbiol. Spectr..

[59] Root Kustritz M.V. (2006). Collection of tissue and culture samples from the canine reproductive tract. Theriogenology.

[60] Barkhoff M., Rojahn A., Siesenop U., Verspohl J., Goericke-Pesch S. (2026). The value of bacteriological culture examination in canine breeding—Are vaginal swab results repeatable?. Reprod. Domest. Anim..

[61] Poulsen C.S., Kaas R.S., Aarestrup F.M., Pamp S.J. (2021). Standard Sample Storage Conditions Have an Impact on Inferred Microbiome Composition and Antimicrobial Resistance Patterns. Microbiol. Spectr..

[62] Panisello Yagüe D., Mihaljevic J., Mbegbu M., Wood C.V., Hepp C., Kyman S., Hornstra H., Trotter R., Cope E., Pearson T. (2021). Survival of *Staphylococcus aureus* on sampling swabs stored at different temperatures. J. Appl. Microbiol..

[63] Tesin N., Stancic I., Tekic D., Acanski A., Kovacevic Z. (2024). Prevalence and antimicrobial resistance trends among vaginal bacteria isolates from pregnant bitches. Reprod. Domest. Anim..

[64] Moerer M., Merle R., Bäumer W. (2022). A Cross-Sectional Study of Veterinarians in Germany on the Impact of the TAHAV Amendment 2018 on Antimicrobial Use and Development of Antimicrobial Resistance in Dogs and Cats. Antibiotics.

[65] Jessen L.R., Sorensen T.M., Lilja Z.L., Kristensen M., Hald T., Damborg P. (2017). Cross-sectional survey on the use and impact of the Danish national antibiotic use guidelines for companion animal practice. Acta Vet. Scand..

[66] Hopman N.E.M., Portengen L., Hulscher M., Heederik D.J.J., Verheij T.J.M., Wagenaar J.A., Prins J.M., Bosje T., Schipper L., van Geijlswijk I.M. (2019). Implementation and evaluation of an antimicrobial stewardship programme in companion animal clinics: A stepped-wedge design intervention study. PLoS ONE.

[67] Pereira A.M., Clemente A. (2021). Dogs’ Microbiome From Tip to Toe. Top. Companion Anim. Med..

[68] Cazer C.L., Lawless J.W., Frye A., Gonzalez L., Greiner Safi A. (2023). Divergent veterinarian and cat owner perspectives are barriers to reducing the use of cefovecin in cats. J. Am. Vet. Med. Assoc..

[69] Hopman N.E.M., Hulscher M., Graveland H., Speksnijder D.C., Wagenaar J.A., Broens E.M. (2018). Factors influencing antimicrobial prescribing by Dutch companion animal veterinarians: A qualitative study. Prev. Vet. Med..

[70] Smith M., King C., Davis M., Dickson A., Park J., Smith F., Currie K., Flowers P. (2018). Pet owner and vet interactions: Exploring the drivers of AMR. Antimicrob. Resist. Infect. Control..

[71] Frey E., Kedrowicz A., Hedgpeth M.-W. (2024). Decision making on antimicrobial use: Cat and dog owners’ knowledge and preferences for veterinary communication. Vet. Rec..

[72] Scarborough R., Hardefeldt L., Browning G., Bailey K. (2021). Pet Owners and Antibiotics: Knowledge, Opinions, Expectations, and Communication Preferences. Antibiotics.

[73] Rocha M.F.G., Paiva D.D.Q., Amando B.R., Melgarejo C.M.A., Freitas A.S., Gomes F.I.F., Ocadaque C.J., Costa C.L., Guedes G.M.M., Lima-Neto R.G. (2022). Antimicrobial susceptibility and production of virulence factors by bacteria recovered from bitches with pyometra. Reprod. Domest. Anim..

[74] Leps A.S., Klein B., Schneider M., Goericke-Pesch S. (2024). How Restrictive Legislation Influences Antimicrobial Susceptibility in Selected Bacterial Isolates from the Canine Vagina. Antibiotics.

[75] Dzieciol M., Nizanski W., Stanczyk E., Kozdrowski R., Najder-Kozdrowska L., Twardon J. (2013). The influence of antibiotic treatment of bitches in oestrus on their attractiveness to males during mating. Pol. J. Vet. Sci..

[76] Delucchi L., Fraga M., Perelmuter K., Cidade E., Zunino P. (2008). Vaginal lactic acid bacteria in healthy and ill bitches and evaluation of in vitro probiotic activity of selected isolates. Can. Vet. J..

[77] Golinska E., Sowinska N., Szydlo M., Witka N., Lenarczyk J., Zbigniew A., Strus M. (2023). The in vitro effects of probiotic bacteria on genital pathogens of female dogs. BMC Vet. Res..

[78] Morales B., Spadetto L., Calvo M.A., Yeste M., Arosemena L., Rigau T., Rivera Del Alamo M.M. (2022). Evaluation of the Probiotic In Vitro Potential of Lactic Acid-Producing Bacteria from Canine Vagina: Possible Role in Vaginal Health. Animals.

[79] Hutchins R.G., Bailey C.S., Jacob M.E., Harris T.L., Wood M.W., Saker K.E., Vaden S.L. (2013). The effect of an oral probiotic containing lactobacillus, bifidobacterium, and bacillus species on the vaginal microbiota of spayed female dogs. J. Vet. Intern. Med..

[80] Socha P.A., Zdunczyk S. (2025). The effect of the oral administration of lactic-acid probiotic bacteria on the vaginal microflora of bitches. J. Vet. Res..

[81] Abt M.C., Pamer E.G. (2014). Commensal bacteria mediated defenses against pathogens. Curr. Opin. Immunol..

[82] Mahiddine F.Y., You I., Park H., Kim M.J. (2023). Management of dog sperm parameters and gut microbiota composition with Lactobacillus rhamnosus supplementation. Vet. Res. Commun..

[83] Hewitt D., England G. (2000). Assessment of optimal mating time in the bitch. Practice.

[84] De Carolis E., Vella A., Vaccaro L., Torelli R., Spanu T., Fiori B., Posteraro B., Sanguinetti M. (2014). Application of MALDI-TOF mass spectrometry in clinical diagnostic microbiology. J. Infect. Dev. Ctries..

